# Three-Loop Technique for Pulley Reconstruction—A Retrospective Cohort Analysis of 23 Patients

**DOI:** 10.3390/jcm12155154

**Published:** 2023-08-07

**Authors:** Simon Oeckenpöhler, Martin Franz Langer, Matthias Michael Aitzetmüller-Klietz, Marie-Luise Aitzetmüller-Klietz, Valerie Nottberg, Oliver Riesenbeck

**Affiliations:** 1 Department of Trauma, Hand and Reconstructive Surgery, University Hospital Münster, Waldeyer Str. 1, 48149 Münster, Germany; simon.oeckenpoehler@ukmuenster.de (S.O.);; 2 Section for Plastic and Reconstructive Surgery, Department of Traumatology, University Hospital Münster, Waldeyer Str. 1, 48149 Münster, Germany; aitzetmueller.m@hotmail.com (M.M.A.-K.); marie.luise.klietz@gmx.de (M.-L.A.-K.); 3 Department of General, Visceral and Thoracic Surgery, University Hospital Hamburg, Martinistrasse 52, 20246 Hamburg, Germany

**Keywords:** pulley reconstruction, flexor pulley, flexor tendon pulley, flexor tendon sheath

## Abstract

Twenty-three patients with a mean age of 52.7 years underwent pulley reconstruction using the Okutsu double- or triple-loop technique after iatrogenic or traumatic rupture of at least two adjacent flexor tendon pulleys in the finger and distal palm; mean age of injury was 4.77 years. The mean follow-up was 4.66 years after reconstruction of mostly A2 pulleys in a single surgeon setting. Outcome measures included ROM, NRS pain, satisfaction, Disabilities of Arm, Shoulder and Hand Questionnaire (DASH) and Krimmer score, Buck–Gramcko score, Jamar grip strength, pinch grip, and vigorimetry compared to the uninjured side. The median patient satisfaction score was 6.6/10. Hand function using the DASH score was 9.5. Grip strength on the Jamar Dynamometer showed only a slight reduction of 13% compared to the uninjured side. The resultant force of the operated fingers on the vigorimeter is almost 60% of that of the contralateral side, and the finger-palm distance of the operated finger was reduced from 2.2 cm to 1.45 cm. Other functional scores, such as Krimmer (82.2) and Buck-Gramcko (10.9), support these good results. The follow-up of patients more than 4.5 years after reconstruction of the A2 and A3 flexor tendon pulley using the double- or triple-loop technique showed acceptable patient satisfaction and good function of the finger in everyday life.

## 1. Introduction

Despite its relatively high prevalence, flexor pulley injury or dysfunction remains underrepresented in the literature. While few studies report clinical outcomes and the long-term follow-up of surgical pulley reconstruction, most studies present surgical descriptions and technical modifications [1,2,3,4,5,6]. Due to the re-popularization of certain sports, such as climbing or bouldering [7,8,9,10], which have a high prevalence of pulley injuries, adequate therapies are needed.

Today, when most studies concentrate on climbers’ injuries, pulley injuries are graded in four levels according to the Pulley Injury Score [8]. While grade I represents a strained pulley, grade II represents either a complete rupture of the A4 pulley or a partial rupture of the A2 or A4 pulleys. Grade III is described as a complete rupture of the A2 or A3 pulley and grade IV is described as combined ruptures or ruptures in combination with injury to the lumbrical muscles or collateral ligaments. While grades I–III can be treated conservatively with immobilization and taping, grade IV injuries require surgical reconstruction.

**Figure 1:**
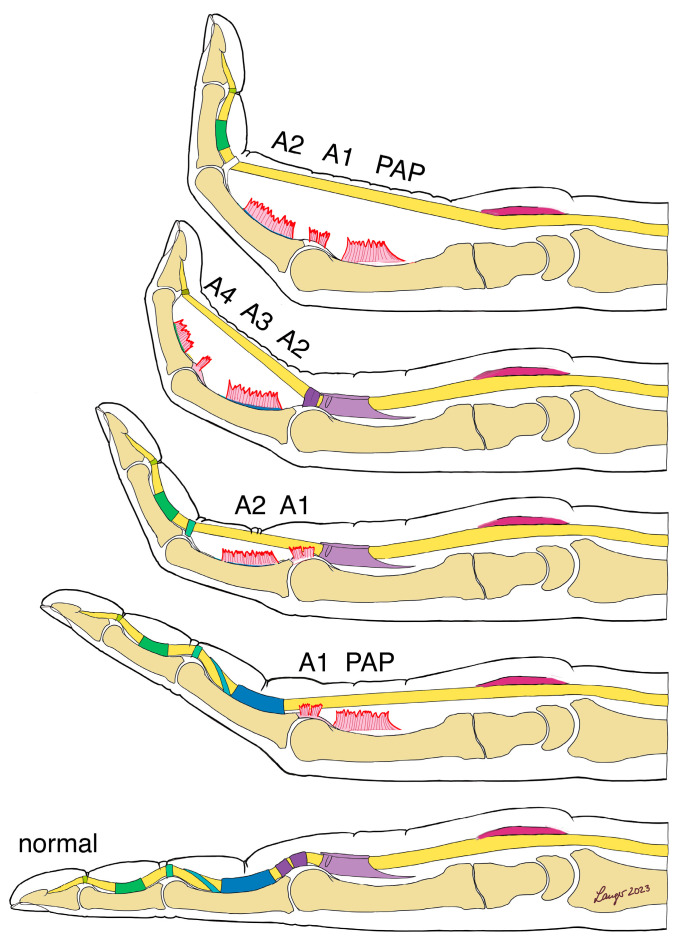
Resulting malfunction in case of insufficiency or injury to the flexor pulley system.

Clinically, the flexor pulleys in combination with the palmar plate are essential for finger flexion. Therefore, patients with rupture or insufficiency of the flexor pulley system present loss of strength or bowstringing, depending on the pulleys involved. Whilst the A1 pulley mainly represents a hypomochleon in extended flexion, the rupture of the A2, A3, and A4 pulleys lead to the compression of the joint and, in the long term, to a flexed position of the finger [11]. A rupture of the A1 pulley usually becomes clinically relevant when the adjacent system is deficient. Combined with a palmar aponeurosis septum defect, it usually results in a contracture of the MCP in a flexed position. A flexion contracture of the PIP is typical of a combined rupture of the A1 and A2 pulleys. If A2, A3, and A4 are insufficient, a bowstringing and flexion contracture will be seen distal to the PIP or, depending on the palmar aponeurosis pulley (PAP), proximal to the MCP (Figure 1). Pulley ruptures that are not treated result in increasing stiffness of the PIP joint with an associated flexion contracture [12]. Over time, this leads to a deterioration in the function of the finger with a corresponding disturbance in the closing of the fist and ultimately to limited function of the hand.

Standardized reports of technical pulley reconstruction are urgently needed to determine the optimal surgical approach for a future cohort of patients. Therefore, we retrospectively analyzed twenty-three (non-climbers) patients who underwent pulley reconstruction using the double- or triple-loop technique with long-term follow-up.

## 2. Materials and Methods

We present a retrospective cohort analysis of twenty-three patients who underwent surgery for flexor pulley insufficiency of the A1, A2, A3 or A4 pulley between 2006 and 2016. All patients were treated by a single surgeon (MFL) using a double- or triple-loop technique [4]. Nine women and fourteen men were treated; mean age at surgery was 52.4 years. The left hand was affected in 13 patients (two left-handed) and the right hand in 10 patients (all right-handed). The cause of insufficiency was hyperextension trauma in two cases, operated M. Dupuytren in two cases, traumatic transection in two cases, and inadvertent iatrogenic dissection of the flexor pulley system in 16 patients. The average time from the original injury or iatrogenic lesion to presentation at our clinic and surgical treatment was 4.77 years.

Two patients underwent reconstruction of the palmar aponeurosis pulley (PAP) and A1 pulley; five patients underwent reconstruction of A1 and A2 pulley; and three patients underwent extended reconstruction of A1, A2, A3, and A4. Seven patients underwent reconstruction of A2–A4 and four patients underwent reconstruction of A1, A2, and A3 pulleys.

Patients were considered for surgery if they had significant limitations in their daily activities and clinical bowstringing. The general function of the flexor tendons had to be intact. Surgery was only indicated if at least two adjacent pulleys were insufficient, or if the A1 pulley and the PASS were insufficient, resulting in a noticeable protrusion of the flexor tendon (Table 1).

**Figure 2:**
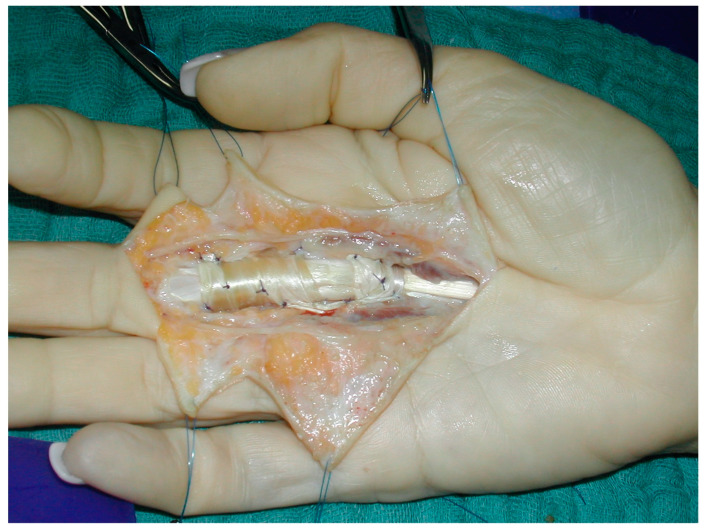
Intraoperative picture after A1 to A3 flexor pulley reconstruction with palmaris longus tendon graft.

All patients were treated using a technique described by Okutsu et al. [4]. Multiple Y-V flaps were used as Bruner’s incisions. Tenolysis of the flexor tendons was performed and, in all cases, a large amount of tough connective tissue under the flexor tendon was resected. In our experience, this connective tissue grows in the course of all clinical bowstringing and increases in size over time, leading to a gradual deterioration in function and is the main reason for flexion contracture and extension deficit. This tight tissue often makes the inexperienced examiner and hand surgeon mistakenly think of Dupuytren’s disease, which presents similarly. In Dupuytren’s disease, the finger cannot be fully extended due to the flexion contracture. In this case, it is essential to find out which disease is present by means of a specific examination. In our experience, the anamnesis is a key factor. Most of the patients in our group can associate the onset of restricted movement with a trauma and, thus, define a starting point. Dupuytren’s disease usually develops insidiously without a clear beginning and is familial. Bowstinging can be clearly detected on sonography where the tendon is clearly separated from the bone and new connective tissue has grown between the tendon and the bone in this later phase of the injury. On the other hand, fresh injuries do not appear to be so easy to detect on ultrasound, as studies on the distance between the tendon and the bone are still unclear as to the distance at which a rupture can be assumed [13]. An MRI provides similar information, but is much more expensive and time-consuming, but has the advantage of providing clear visualization at a very early stage [14]. In the clinical examination, only a central cord corresponding to the flexor tendon can be palpated and usually no nodular alterations. In some cases, the redressing of the tendon to the bone is also partially possible, which leads to better active flexion. In Dupuytren’s disease, flexion is usually only slightly limited; however, in bowstringing, it is always limited. After the resection, the flexor tendon can be pulled down directly onto the bone as it had a significant gap before the tissue resection. The palmaris longus tendon was exposed through an oblique 10 mm incision proximal to the palmar flexion crease and harvested with a tendon stripper. The harvested tendon was then wrapped around the proximal phalanx and flexor tendon two or three times depending on the extent and length of the injury. Tension was then adjusted with sutures to allow the tendon to slide under passive pressure on the forearm and not lift off the bone during flexion. The graft was fixed to the palmar plate of the MP, PIP, or DIP joint with 4-0 PDS. Additional sutures were placed between the tendon graft strips to further stabilize the reconstruction. After adequate hemostasis, skin closure was performed. V-Y flap plasty can be made through the initial Brunner’s incisions during skin closure. Usually, tension of the skin is reduced due to the relocation of the tendon to the bone and the resection of the connective tissue and an excess of skin is present. The final operative result is shown in Figure 2.

Postoperatively, the fingers are placed in a splint in straight position for four weeks. Physiotherapy with manual palmar pressure with active and passive motion was started three days after surgery. Manual work and sports with the affected hand were avoided for at least two months and powerful grips for 3 months in all patients.

The follow-up was conducted by an investigator (VN) who was not involved in the surgery or in the postoperative care and follow-up. The data for evaluation were collected at one point in time, which was a median of 4.66 years after the surgical reconstruction.

ROM was recorded for flexion and extension of the injured finger for each joint using a goniometer. Grip strength in both hands was measured using a Jamar hydraulic hand dynamometer (Performance Health Supply, Cedarburg, WI, USA) and a mechanical pinch gauge (Baseline Evaluation Instruments, White Plains, NY, USA) and a Martin Vigorimeter [15]. The peculiarity of the strength measurement of these patients is the type of grasping that develops as a result of the contracture. On the one hand, larger objects, such as the Jamar dynamometer, cannot be grasped with the finger because the affected finger cannot be fully extended. On the other hand, the force at the end of the fist closure is also altered. Therefore, we used the Martin Vigorimeter in such a way that only the affected finger alone pressed on the balloon of the Vigorimeter. This made it possible to measure and objectify the terminal force during fist closure, which, according to our information, is not adequately tested by any other measuring instrument. The force in maximum flexion is relevant for the stable holding of small objects, such as ropes, so that there can be a high relevance for specific activities. The Numeric Pain Scale (NPS; range 0–10), DASH score [16], Krimmer score [17], and Buck–Gramcko score [18] were also recorded. Overall subjective satisfaction was measured using a visual analogue scale, with a score of 10 representing maximum satisfaction.

Statistical analysis was performed using IBM SPSS Statistics 25 and Graphpad Prism 7.0. Following the Kolmogorov–Smirnov test, a two-tailed dependent *t*-test was performed to assess statistical differences. All data are presented as mean ± standard error of the mean (SEM). A *p*-value of 0.05 was considered statistically significant.

## 3. Results

Postoperative complications occurred in 3 patients (13%) and were graded according to the Clavien–Dindo classification [19]. Two patients reported a manifestation of a neuroma and mild numbness, which corresponds to a grade I complication. One patient developed a grade III complication and underwent reoperation due to adhesions of the flexor tendon.

### 3.1. Numeric Pain Scale, DASH Score, Krimmer Score and Satisfaction

The mean NPS was 1.2 ± 0.41, with a minimum of 0 and a maximum of 6. The DASH score of the operated hand was 16.03 ± 3.48. The mean Krimmer score was 82.17 ± 18.27, representing a satisfactory result. In addition, mean postoperative satisfaction was 6.6 ± 0.66 (0 being unsatisfied and 10 being maximally satisfied), see also Table 2.

### 3.2. Dynamometry

The surgically treated hand showed a mean grip strength of 29.28 kg ± 2.71 on the Jamar dynamometer, which was significantly lower than the non-operated hand (33.45 kg ± 2.68, *p* = 0.00025). 

The pinch test of the operated finger showed a mean strength of 3.46 kg ± 0.36 compared to 4.17 kg ± 0.67, showing no significant difference (*p* = 0.26). 

Martin Vigorimeter showed a mean strength of the reconstructed finger of 15.02 kPa ± 2.85 compared to 25.9 ± 3.25 of the unaffected side, which was significantly improved (*p* < 0.001), see also Table 2.

**Table 2:** Overview of results Section 3.1 and Section 3.2.

	Operated Side	Non-Operated Side
Numeric pain scale	1.2 ± 0.41	13° ± 5°
DASH	16.03 ± 3.48	51° ± 5°
Krimmer Score	82.17 ± 18.27	69° ± 5°
Satisfaction (0–10 points)	6.6 ± 0.66	
Grip strength	29.28 kg ± 2.71	33.45 kg ± 2.68
Pinch strength	3.46 kg ± 0.36	4.17 kg ± 0.67
Martin Vigorimeter	15.02 kPa ± 2.85	25.9 ± 3.25

### 3.3. Movement

ROM was tested separately for each joint and compared to preoperative measurements and contralateral measurements. ROM data for the DIP, PIP and MCP joints are shown in Table 3. 

Active extension in the DIP joint showed a deficit of 10° ± 4° preoperatively, 6° ± 2° postoperatively and 5° ± 3° in the untreated side. We found no significant difference between pre- and postoperative or between operated and unoperated hand in the movement of the DIP (*p* = 0.178 and *p* = 0.817, respectively). The flexion of the DIP was 23° ± 6° preoperatively and 57° ± 6° postoperatively, showing a significant improvement (*p* < 0.001).

Active extension in the PIP joint showed a deficit of 25° ± 6° in lacerated fingers, 10° ± 4° postoperatively, and 3° ± 1° in uninjured fingers, showing a significant improvement with flexor pulley reconstruction (*p* = 0.001) and no significant difference between reconstructed and uninjured fingers (*p* = 0.099). Preoperative flexion was 51° ± 7°, 69° ± 6° postoperatively, and 86° ± 2° in healthy fingers. Significant differences were found between pre- and postoperative and between postoperative and healthy fingers (*p* = 0.001 and *p* = 0.002). 

The MCP showed a deficit in extension of 19° ± 5° preoperatively; 11° ± 4° in the treated finger, which was significantly improved (*p* = 0.004); and 1° ± 1° in the unaffected fingers. Active flexion in the MCP was 72° ± 5° preoperatively, 84° ± 2° in the treated finger, and 87° ± 2° in the unaffected fingers, showing no significant efficacy of surgery (*p* = 0.009) and no significant difference between the reconstructed and unaffected fingers (*p* = 0.32). 

**Figure 3:**
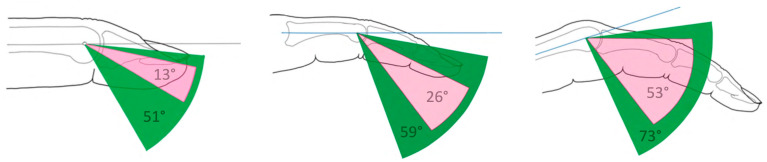
Schematic movement of DIP, PIP, and MCP (**left**, **middle**, and **right**) preoperatively (pink) and postoperatively (green) shows the additional ROM after pulley reconstruction.

The total range of motion (in °) is shown in Table 3 and Figure 3.

### 3.4. Fingertip to Palm Distance

Fingertip to palm distance was measured pre- and postoperatively in 21 patients. Unfortunately, preoperative data are missing for two patients. The mean preoperative fingertip-to-palm distance was 28 mm ± 2 mm. Postoperative measurements show a mean distance of 16 mm ± 2 mm, which is significantly reduced (*p* = 0.004).

## 4. Discussion

Although the importance of at least two functional pulleys has been well-proven in biomechanical and clinical studies [20], the evaluation of surgical treatment has been poorly addressed. Bowers et al. reported the outcome of late diagnosed, conservatively and surgically treated flexor pulley insufficiency, but surgical technique and postoperative measurements were poorly described [21]. They concluded that surgical reconstruction should be performed in patients with limited ROM, bowstringing, and increased pain, which was confirmed by Moutet in 2003 [22]. 

Bollen et al. retrospectively analyzed 67 professional climbers with annular pulley ruptures treated conservatively with immobilization or taping resulting in flexion contracture or PIP joint insufficiency [23,24].

Current surgical techniques differ in terms of the graft used and the fixation technique. Fritz et al. showed that the revascularization properties and perhaps also the sliding properties of tendons within a tendon sheath, such as the superficial flexor tendons, correspond anatomically better to the flexor pulleys. However, the elevation morbidity of a superficial flexor tendon is, in our opinion, so massive compared to the palmaris longus that we continue to recommend the palmaris longus tendon as a primary graft. To date, clinical studies are lacking and the data take into account electronic microscopy, histology, and immunohistochemistry, but not clinical characteristics [25].

In 2007, Arora et al. [26] reconstructed 23 annular pulleys by using a technique originally described by Kleinert et al. [1]. The use of the palmaris longus tendon or the extensor retinaculum has also been described by Gabl [27]. Both techniques were found to be equally effective for PIP flexion (81.5° preoperative to 91° postoperative). The pinch test was comparable between techniques. Although our population had a more severe stage, we were able to improve PIP flexion by 18° (from 51° ± 5° to 69° ± 5° postoperatively). 

Arora mainly treated sports climbers, patients who usually injured the A2 or A3 flexor pulley, resulting in a relatively short defect. Our population was heterogeneous, ranging in age from eleven to eighty-five years, with different patterns of flexor pulley injury. It is, therefore, difficult to make direct comparisons, especially as the patients we described all had an old injury and, therefore, already had pronounced sub-tendinous scarring.

Okutsu et al. found a mean improvement of 30° in the PIP, DIP, and MCP joints (preoperative 175° compared to 205°) and a reduction in fingertip-to-palm distance from 32 mm to 22 mm [4]. In our population, we found a mean ROM improvement of 91° with reconstruction (92° compared to 183°). In addition, the fingertip-to-palm distance in our population was 28 mm preoperatively and 16 mm postoperatively. Therefore, the efficacy of surgery appears to be comparable.

While many currently available studies use single measurements to evaluate techniques, we used a range of postoperative outcome measures. Using the Jamar dynamo-meter, pinch test and Martin Vigorimeter, we were able to measure grip strength as well as isolated dynamic and static strength of the affected fingers. We also measured values for both the healthy and affected side to eliminate individual factors. Scores such as the DASH or Krimmer scores are widely used to characterize loss of limb function and limitations in daily life. 

We measured flexibility by evaluating the fingertip-to-palm distance and by using goniometry separately for the joints. All values showed significant improvement with surgery.

Our study population can be described as relatively heterogeneous, with no sports climbers included and the above-mentioned age range. In addition, the time from initial injury to surgery was relatively long, with an average of 4.77 years. Most of the other studies included only patients with a recent injury. Therefore, fibrotic adhesions and scarring between the bone and tendon influenced outcomes and resulted in worse preoperative ROM compared to similar studies [1,2,3,4,5,6,22]. Nevertheless, surgical intervention was effective in improving ROM, reducing pain, and improving DASH and Krimmer scores. It should be emphasized that the still active progression of finger dysfunction, which occurs in all cases of persistent insufficiency, was halted, stopped, and reversed by surgery. 

## 5. Conclusions

Reconstructions of the flexor pulleys have been described in the literature since 1931. Various authors have described and presented different techniques, none of which, to the best of our knowledge, have prevailed over the others as evidenced by facts and studies to date. Few studies have shown the objective results in the medium term after such an intervention in a patient population with an older injury, so comparability is poor. The study presented here shows the objective results after reconstruction with the Okutsu technique with many outcome parameters that can be used for further studies to compare data. In our opinion, this can be considered a safe and efficient technique in a very heterogeneous patient population, leading to improved overall hand function even in complex, overlooked, and untreated injuries with their complications of scarring.

## Figures and Tables

**Table 1:** Inclusion and exclusion criteria for surgery.

Inclusion Criteria	Exclusion Criteria
Clinical bowstringing	M Dupyutren
Loss of grip strength	Loss of sensation in the finger
Cognitive understanding for aftercare	Flexor tendon rupture
Protrusion of the flexor tendon	Extensive scarring over the tendons

**Table 3:** Comparison of total range of motion of the fingers in detail: pre-, postoperatively, and contralateral (non-operated) side (rounded values to whole degrees).

Joint	Group	Total ROM	Extension-Lag	Flexion
DIP	preoperative	13° ± 5°	10° ± 4°	23° ± 6°
DIP	postoperative	51° ± 5°	6° ± 2°	57° ± 6°
DIP	contralateral	69° ± 5°	5° ± 3°	64° ± 2°
PIP	preoperative	26° ± 5°	25° ± 6°	51° ± 7°
PIP	postoperative	59° ± 5°	10° ± 4°	69° ± 6°
PIP	contralateral	83° ± 4°	3° ± 1°	86° ± 2°
MCP	preoperative	53° ± 7°	19° ± 5°	72° ± 5°
MCP	postoperative	73° ± 5°	11° ± 4°	84° ± 2°
MCP	contralateral	86° ± 4°	1° ± 1°	87° ± 2°

## Data Availability

The data are not publicly available due to privacy of the included patients.
